# Shared genetic loci and causal relations between schizophrenia and obsessive-compulsive disorder

**DOI:** 10.1038/s41537-023-00348-x

**Published:** 2023-04-07

**Authors:** Yu Chen, Hua Guo, Weihua Yue

**Affiliations:** 1 Department of Psychiatry, The Second Affiliated Hospital of Xinxiang Medical University, Xinxiang, Henan 453002 China; 2 Zhumadian second people’s hospital, Henan, 463899 China; 3 Institute of Mental Health, Peking University Sixth Hospital, Beijing, 100191 China; 4 NHC Key Laboratory of Mental Health (Peking University), National Clinical Research Center for Mental Disorders (Peking University Sixth Hospital), Beijing, 100191 China; 5 PKU-IDG/McGovern Institute for Brain Research, Peking University, Beijing, 100871 China; 6 Research Unit of Diagnosis and Treatment of Mood Cognitive Disorder (2018RU006), Chinese Academy of Medical Sciences, Beijing, 100191 China

**Keywords:** Schizophrenia, Biomarkers, Target identification, Genetics of the nervous system

## Abstract

Based on the clinical overlap between schizophrenia (SCZ) and obsessive-compulsive disorder (OCD), both disorders may share neurobiological substrates. In this study, we first analyzed recent large genome-wide associations studies (GWAS) on SCZ (*n* = 53,386, Psychiatric Genomics Consortium Wave 3) and OCD (*n* = 2688, the International Obsessive-Compulsive Disorder Foundation Genetics Collaborative (IOCDF-GC) and the OCD Collaborative Genetics Association Study (OCGAS)) using a conjunctional false discovery rate (FDR) approach to evaluate overlap in common genetic variants of European descent. Using a variety of biological resources, we functionally characterized the identified genomic loci. Then we used two-sample Mendelian randomization (MR) to estimate the bidirectional causal association between SCZ and OCD. Results showed that there is a positive genetic correlation between SCZ and OCD (r_g_ = 0.36, *P* = 0.02). We identified that one genetic locus (lead SNP rs5757717 in an intergenic region at CACNA1I) was jointly associated with SCZ and OCD (conjFDR = 2.12 × 10^−2^). Mendelian randomization results showed that variants associated with increased risk for SCZ also increased the risk of OCD. This study broadens our understanding of the genetic architectures underpinning SCZ and OCD and suggests that the same molecular genetic processes may be responsible for shared pathophysiological and clinical characteristics between the two disorders.

## Introduction

Despite schizophrenia (SCZ) and obsessive-compulsive disorder (OCD) being regarded as separate and rarely overlapping diagnostic entities, these disorders apparently share high comorbidity: the rate of SCZ with obsessive-compulsive symptoms comorbidity was up to 30%^1^, SCZ with OCD (schizo-obsessive comorbidity, SOC) 12–14%^2^, schizotypal personality with OCD 12–14%^3^. A prior diagnosis of OCD was associated with higher rates of schizophrenia and schizophrenia spectrum disorders later in life, after adjusting for a family history of psychiatric disorders and the patient’s psychiatric history^4^. Adolescent, adult, and elderly patients with schizophrenia all displayed obsessive-compulsive symptoms (OCS), providing more evidence that OCD and schizophrenia may be related^5,6^. OCS first appeared while taking atypical antipsychotic medications to treat schizophrenia, which raised the possibility that comorbid OCD was a side effect of medication. But comparable comorbidity rates of OCS were reported among individuals at ultrahigh risk for psychosis^7–9^, in prodromal phases of schizophrenia^10–12^, and in drug-naive patients with first-episode schizophrenia^8,13^. Therefore, OCS in schizophrenia cannot be a result of antipsychotic medication or a chronic illness. More studies are needed to explore the temporal relationship between two disorders in a longitudinal design.

Additionally, an increasing number of translational, neurophysiological, and neuroimaging studies suggest a substantial overlap in the pathophysiology of SCZ and OCD. Both SCZ and OCD showed dissociable source-monitoring impairments and oculomotor function deficits; only in SCZ does a specific deficiency in the motor control of saccades, smooth eye pursuit, and reality monitoring and OCD patients showed impairment in the maintenance of active fixation, suggesting that SCZ and OCD have shared and specific neurobiological substrates^14,15^. The shared polygenic risk between SCZ and OCD was detected in a cross-disorder polygenic risk score analysis^16^. A similar pattern of accelerated white matter decline with age and greater white matter deficit in females in OCD and SCZ: small fractional anisotropy (FA) reductions in the corpus callosum and accelerated reductions in FA with age while OCD having specifically in the left superior longitudinal fasciculus and SCZ showing a more widespread pattern of FA reduction^17^. A key neurological defect of mental diseases has been postulated to be aberrant functional integration within the default mode network (DMN) in SCZ and OCD^18^. Functional connectivity (FC) between the subregions of DMN increased in SCZ, SOC, and OCD compared with the healthy control group, while SOC and SCZ showed significantly increased FC between DMN subregions and middle temporal gyrus but OCD exhibited decreased FC^19^. Taken together, the previous findings suggest that SCZ and OCD share neurological foundations for them.

More recently, genome-wide association studies (GWAS) have demonstrated that schizophrenia is a polygenic psychiatric disorder, the SNP-based heritability was estimated to be 0.24 (s.e. = 0.007)^20^. The common SNP heritability in the largest single OCD genome-wide study (combined OCGAS and IOCDF-GC) was estimated to be 0.28 (s.e. = 0.04)^21^. Still no evidence for genes underline the genetic correlation between SCZ and OCD, there was some clues for genetic disposition. The gene SLC1A1 (solute carrier family) on chromosome 9p24 was associated with OCS in schizophrenia but not with vulnerability to schizophrenia^22^. While other studies found the gene DLGAP3 (disks large associated protein 3)^23^ and GRIN2B (the type 2B subunit of the N-methyl-D-aspartate receptor)^24^ may interact with SLC1A1 for OCS in schizophrenia. Besides, Val66Met polymorphism in brain-derived neurotrophic factor (BDNF) was associated with OCS in schizophrenia^25^. Although there are many studies investigating the relationships between SCZ and OCD, the relationship between the two is complex and unclear. We hypothesize that SCZ and OCD have a common genetic basis. Here, we applied MiXeR^26^ and conjFDR analysis^27–29^ to the latest GWASs to assess the shared genetic basis between SCZ and OCD in European populations. MiXeR was used to quantify polygenic overlap beyond genetic correlation. Further, we investigate if there are genetic loci jointly associated with SCZ and OCD using the cond/conjFDR method. We also conducted a Mendelian randomization (MR) approach to estimate the bidirectional association between SCZ and OCD.

## Results

### Genetic correlation

**Fig. 1:**
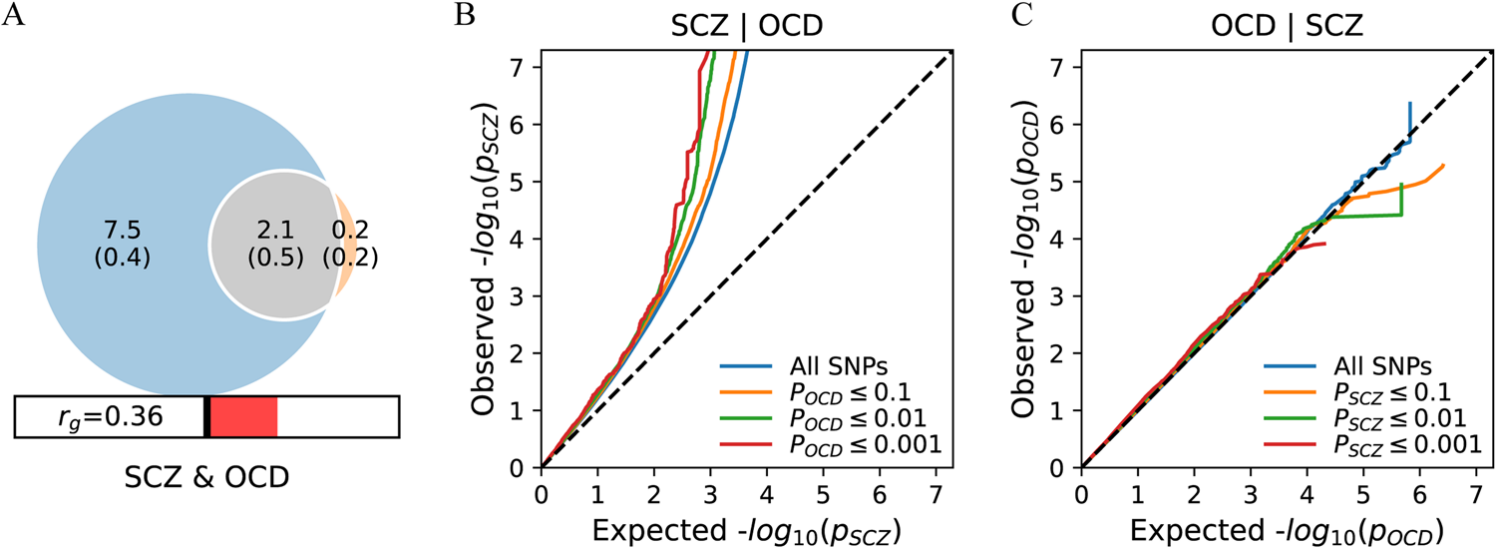
Polygenic overlap between SCZ and OCD. **A** The polygenic overlap between SCZ (blue) and OCD (orange). Venn diagrams of causal shared and unique variants. The numbers is the quantity of causal variants with standard errors in parentheses (numbers in thousand). Conditional Q-Q plots of nominal versus empirical −log10 *p* values (corrected for inflation) in **B** SCZ below the standard GWAS threshold of *P* < 5 × 10^−8^ as a function of significance of association with OCD, and **C** vice versa at the level of *P* < 0.1, *P* < 0.01, *P* < 0.001, respectively. The blue lines indicate all SNPs. SCZ schizophrenia, OCD obsessive-compulsive disorder r_g_; genetic correlation.

We found a positive genetic correlation between SCZ and OCD (r_g_ = 0.36, *P* = 0.02) (Fig. 1A).

### Polygenic overlap

Univariate MiXeR estimated that 9600 trait-variants influenced SCZ and 2300 variants influenced OCD. Bivariate MiXeR analysis revealed a substantial polygenic overlap of SCZ-influencing variants OCD. Of the 9600 SCZ-influencing variants, 2100 (SD = 500) were also predicted to influence OCD (Fig. 1A). In Conditional Q-Q plots, We observed a pronounced successive leftward deflection for SCZ | OCD (Fig. 1B), but not for OCD | SCZ (Fig. 1C), indicating polygenic enrichment and a potential causal relationship.

### SCZ/OCD-associated loci

Using condFDR we identified 256 (Table S1) LD-independent loci to be significantly (condFDR < 0.01) associated with SCZ after conditioning on association with OCD. No loci were found for OCD after conditioning on SCZ, indicating a unidirectional causality.

### Shared loci

**Fig. 2:**
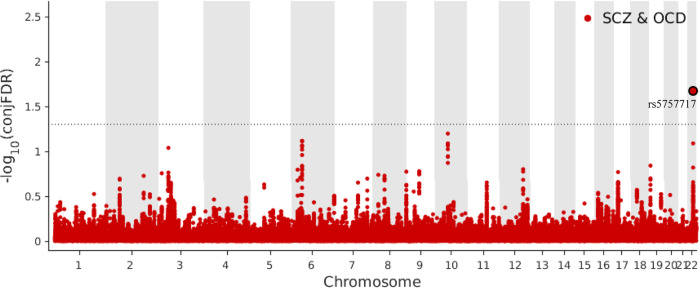
ConjFDR Manhattan plot. Common genetic variants both associated with SCZ and OCD at conjunctional false discovery rate (conjFDR) < 0.05. Manhattan plot showing the −log10 transformed conjFDR values for each SNP on the y axis and the chromosomal positions along the x axis. The dotted horizontal line represents the threshold for significant shared associations (conjFDR < 0.01, i.e. −log10(conjFDR) > 2.0). Independent lead SNPs are encircled in black, and labeled by its nearest gene. SCZ schizophrenia, OCD obsessive-compulsive disorder.

To identify the genetic loci jointly associated with both SCZ and OCD we used conjFDR. We found only one genetic locus jointly associated with SCZ and OCD in an intergenic region at CACNA1I (lead SNP rs5757717, chromosome 22: 39942234, *P* value SCZ = 3.38 × 10^−5^, *P* value OCD = 2.58 × 10^−5^, conjFDR = 2.12 × 10^−2^, Fig. 2). As denoted by the sign of the effect sizes, this loci has consistent effect directions in SCZ and OCD, indicating that the respective risk alleles are linked to higher susceptibility to both SCZ and OCD (Table 1).

**Table 1:** Genomic loci jointly associated with SCZ and OCD at conjunctional FDR < 0.05.

Locus	CHR	Lead SNP	A1/A2	Nearest Gene	Functional category	*P* value OCD	Z-score OCD	*P* value SCZ	Z-score SCZ	conjFDR
1	22	rs5757717	T/C	CACNA1I	Intergenic	2.58E-05	−4.21	3.38E-05	−4.15	2.12E-02

The most strongly associated SNPs in independent genomic loci shared between SCZ and OCD at conjFDR<0.05 after merging regions < 250 kb apart into a single locus. The table presents chromosomal position (CHR), nearest gene and functional category, as well as *p*-values and z-scores from the original summary statistics on SCZ and OCD. The effect sizes are given with reference to allele 1 (A1).

**Fig. 3:**
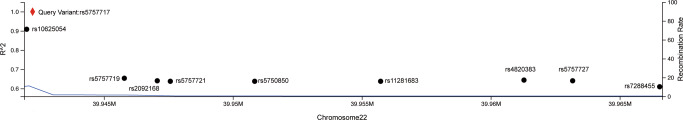
Comprehensive panel displaying LD of query variant (rs5757717). LD information was showed from the 1000 Genomes Phase 3 for European-ancestry haplotype reference panel using an R^2^ cutoff of 0.6 within 250 kb.

**Fig. 4:**
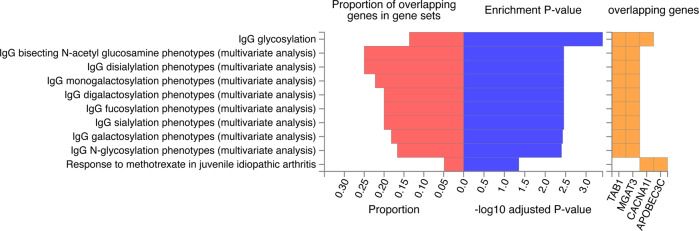
Gene sets associated with the reported genes from the GWAS-catalog. Hypergeometric tests are performed to test if genes of interest are overrepresented in any of the pre-defined gene sets. Multiple test correction is performed.

We applied FUMA to candidate SNPs (*r*^*2*^ ≥ 0.6 with lead SNP, see Fig. 3, Supplemental Table 2) to 11 protein-coding genes (see Supplemental Table 3). Positional mapping aligned 2 SNPs to CACNA1I, cis-eQTL mapping implicated 2 genes (CBX7 and MGAT3), and chromatin interaction mapping implicated 8 genes (APOBEC3C, TAB1, MIEF1, ADSL, SGSM3, MCHR1,PHF5A and ACO2). A gene-set analysis identified no biological process significantly associated with the 11 genes, but these genes are involved in IgG glycosylation (TAB1, MGAT3, and CACNA1I) and Response to methotrexate in juvenile idiopathic arthritis (CACNA1I and APOBEC3C) reported by GWAS catalog (see Fig. 4).

### Mendelian randomization

**Fig. 5:**
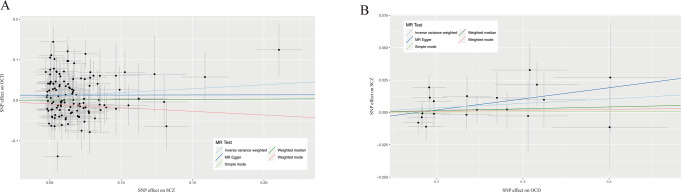
MR plots for the relationship between SCZ and OCD. **A** Scatter plot of SNP effects on SCZ versus OCD, **B** Scatter plot of SNP effects on OCD versus SCZ, with the slope of each line corresponding to the estimated MR effect per method. The data are expressed as raw β values with 95% CIs.

The inverse-variance weighted method showed that SCZ was associated with an approximately 21% increased risk of developing OCD (IVW OR = 1.25; 95% CI, 1.06 to 1.38; *P* = 0.005; see Table 2, Fig. 5). The MR-Egger regression revealed that horizontal pleiotropy was unlikely to bias the result (Egger_intercept = 0.01, *P* = 0.48). The Cochran’s Q test indicated some heterogeneity among the IVs (*P*_*IVW*_ = 1.62 × 10^−6^). After removing the outliers (rs10861176 and rs12877581) detected by MR-PRESSO (β_*raw*_ = 0.20, S.D. = 0.06, *P* = 0.001), robust evidence genetically yielded consistent directions and similar effect estimates (β_*Outlier-corrected*_ = 0.20, S.D. = 0.06, *P* < 0.001). The statistical power is sufficient (0.91) assuming the true causal OR of SCZ on OCD is 1.21, given the involved sample size and the significance level α as 0.05.

**Table 2:** MR results for the relationship between SCZ and OCD.

Method	Number of SNPs	OR (95% CI)^a^	*P*
SCZ on OCD			
MR Egger	128	1.00 (0.59 to 1.72)	0.99
Weighted median		1.01 (0.87 to 1.19)	0.85
Inverse variance weighted		1.21 (1.06 to 1.38)	**0.004**
Simple mode		0.83 (0.51 to 1.36)	0.47
Weighted mode		0.83 (0.52 to 1.33)	0.44
OCD on SCZ			
MR Egger	19	1.04 (0.98 to 1.11)	0.19
Weighted median		1.01 (0.98 to 1.04)	0.65
Inverse variance weighted		1.02 (0.99 to 1.04)	0.13
Simple mode		1.00 (0.95 to 1.06)	0.97
Weighted mode		1.00 (0.96 to 1.05)	0.87

Indicates the odds for SCZ per one-s.d. increase in OCD. All statistical tests were two-sided. *P* < 0.05 was considered significant.

Bold values denote statistically significant results.

The univariable MR estimates were not statistically significant for the effect of OCD (IVW OR = 1.02; 95% CI, 0.99 to 1.04; *P* = 0.13) on SCZ. While the statistical power is insufficient (0.07) assuming the true causal OR of SCZ on OCD is 1.02, given the low sample size. MR-Egger intercept analysis found no evidence of directional pleiotropy (*P* = 0.39). The Cochran’s Q statistic indicated no evidence for pleiotropy (*P*_*IVW*_ = 0.87) across instrument effect. MR-PRESSO analysis did not identify significant horizontal pleiotropic variants and the causal association between serum OCD and SCZ was still observed.

## Discussion

This study provided that there was a large number of trait-influencing variants between SCZ and OCD, as revealed by the MiXeR analyses. while condFDR methods found that no loci were found for OCD after conditioning on SCZ, this could be due to the small sample size of the OCD GWAS dataset.MR analyses suggested that higher genetically-predicted SCZ was associated with the risk of OCD. We added evidence of shared genetic architecture between SCZ and OCD and one shared genetic loci showed a consistent pattern of effect direction for both traits. A gene-set analysis indicated that IgG glycosylation (TAB1, MGAT3, and CACNA1I) and Response to methotrexate in juvenile idiopathic arthritis (CACNA1I and APOBEC3C) may engage common underlying genetic vulnerabilities. These findings provide new knowledge about the relationship and shared genetic mechanisms between SCZ and OCD.

In the present study, we first discovered a genetic overlap between SCZ and OCD, a positive genetic correlation. A meta-analysis of 43 studies with 3978 subjects reported the mean prevalence of OCD to be 12.3%^1^. A clinical study reported that 19% of 200 hospitalized patients with schizophrenia meet the Diagnostic and Statistical Manual of Mental Disorders-IV (DSM-IV) for OCD^30^. Most opinions believe that antipsychotics induced OCS or OCD. Secondary OCS can occur in up to 70% of schizophrenia patients taking second-generation antipsychotics (SGA) like clozapine, olanzapine, or risperidone^31^. Furthermore, a longer duration of treatment with SGAs was related to a higher prevalence of denovo OCS^32^. However, there have been mixed findings regarding atypical antipsychotics and the risk of OCD. In contrast to the above findings, people with schizophrenia spectrum disorders have also reported some positive effects of olanzapine or risperidone on OCS^33,34^. Additionally, there is strong data demonstrating that augmentation of serotonin reuptake inhibitors, olanzapine or risperidone, can lessen OCS in individuals with treatment-resistant OCD^35,36^. These paradoxical effects on OCS may be partially explained by the common genetic factors for both mental disorders. The solute carrier family 1 member 1 (SLC1A1) gene has been consistently associated with primary OCD patients and the presence of the same SNP has been shown to increase the risk of OCS/OCD in schizophrenia with antipsychotic medications^37,38^. Recent studies have found that the incidence in patients with FEP was noted to range from 9.1% to 10.6% for OCD^39^. Overall, an affinity between OCD and schizophrenia is supported by many studies, future studies should recruit unmedicated patients with schizophrenia and OCD to uncover the potential pathogenesis of the comorbidity.

Second, we found that lead SNP rs5757717 in an intergenic region at CACNA1I was jointly associated with SCZ and OCD. The CACNA1I gene, involved in calcium signaling in neurons, is a member of a subfamily of calcium channels referred to as a low voltage-activated, T-type, calcium channel. Many large-scale genetic association studies reported that CACNA1I was implicated in the susceptibility to schizophrenia^40,41^. CACNA1I was considerably up-regulated in the hippocampus of SCZ patients compared to controls, according to RNA-seq analysis, suggesting that dysregulation of CACNA1I may play a role in the etiology of schizophrenia^42^. And unfortunately, the GWAS study for OCD did not yield significant results for the sample size was relatively low compared to many GWAS and replication studies. Evidence from earlier studies indicated that an escalating dose regimen of a Ca channel blocker (CCB) significantly worsened the OCD while cutting back on the CCB dosage improve OCD symptoms^43^. Animal studies suggest that calcium-channel antagonists (flunarizine, amlodipine, etc.) significantly inhibited marble-burying behavior (an animal model of obsessive-compulsive disorder) in mice^44^ and Citalopram, a long-term antidepressant, disinhibited the serotonergic spontaneous firing activity by decreasing the gamma-aminobutyric acid B receptor-mediated regulation of L-type voltage-dependent Ca2+ channels in serotonergic neurons^45^. In a word, there is no evidence supporting the direct connection between CACNA1I and OCD, but Calcium Signaling Pathway may be involved in the pathogenesis and treatment of OCD. We also found that the genes mapped by candidate SNPs were involved in IgG glycosylation reported by GWAS catalog. There are relatively few studies on this area. Many studies showed patients with schizophrenia had a significant increase in circulating IgG levels and a pro-inflammatory IgG receptor in the midbrain^46,47^ while serum toxoplasma IgG positivity was correlated with a greater risk of OCD^48^. This points that the alteration of IgG glycosylation could be a signature of a comorbid phenotype shared by SCZ and OCD, possibly due to genetic basis.

Last, Mendelian randomization results showed that SCZ is a causal risk factor for OCD. Most opinions believe that a diagnosis of OCD confers a risk for later development of schizophrenia and schizophrenia spectrum disorders^4^. But drugs with predominant anti-serotonergic profile (clozapine and risperidone) not predominant dopaminergic blockade (amisulpride and aripiprazole) were responsible for inducing OCS in SCZ^49^ and chronic use of clozapine is also known to downregulate hypersensitive 5-HT2C receptors^50^. Moreover, polymorphisms and interactions between SLC1A1, DLGAP3, GRIN2B and BDNF were associated with antipsychotic-induced OCS in SCZ, suggesting genetic vulnerabilities to both SCZ and OCD. The MR results of present study could be due to the small sample size of OCD GWAS and limited statistical power. On the other hand, only a small portion of the population had schizo-obsessive comorbidity (SOC), similar to OCD and SCZ, indicating a distinct brain disorder. Based on imaging evidence, SOC showed the highest rsFC strength within subregions of the DMN and the lowest rsFC strength between the Default Mode Network (DMN)^19^, changed connection probability of white matter in the DMN, and the Visual Network, the Somatosensory Network^51^ and altered grey matter volume and cortical thickness compared with SCZ, OCD, and healthy control^52^. In real clinical practice, there exist a set of patients having both SCZ and OCD, partially regulated by genetic factors.

The present study uses the conjFDR statistical framework to boost statistical power and uncover polygenic overlap between SCZ and OCD, which may be useful for elucidating the mechanism of comorbidity from genetic and phenotypic perspectives. Limitations include that the sample size of the OCD GWAS study is relatively low and many loci may have been missed for limited statistical power.

## Conclusion

The present study identified shared genetic architecture beyond the genetic correlation between SCZ and OCD. Mendelian randomization results showed that SCZ is a causal risk factor for OCD. We also revealed that CACNA1I gene shared the genetic basis of two disorders, suggesting that genetic factors cause some of the comorbidity for SCZ and OCD.

## Methods

### GWAS summary data sets

The GWAS data on SCZ was obtained from the Psychiatric Genomics Consortium (PGC). Because of potential genetic differences, we included 53,386 cases and 77,258 controls in the European population. Summary statistics for OCD were acquired from the two consortia, the International Obsessive-Compulsive Disorder Foundation Genetics Collaborative (IOCDF-GC) and the OCD Collaborative Genetics Association Study (OCGAS), yielding a total of 2688 individuals of European ancestry with OCD and 7037 matched controls. The relevant ethics committees have approved all of the GWAS data sets used in this investigation, and all subjects gave their informed consent. The original publications provide more details on the requirements for inclusion.

### Genetic correlation

We used LD score regression to estimate genetic correlations (*r*_*g*_) between SCZ and OCD. The sign of a genetic connection, which ranges from 1 to +1, denotes whether the same genetic variants are driving variation in the same or opposing directions.

### Polygenic overlap analysis

MiXeR was used to quantify polygenic overlap between SCZ and OCD irrespective of the effect directions and coefficients. We performed 20 iterations using 2 million randomly selected SNPs for each iteration after random pruning at a linkage disequilibrium threshold of *r*^*2*^ = 0.8. The Venn diagram was displayed to count the number of shared and trait-unique causal variants between two traits. The presence of pleiotropic enrichment was determined by an increasing leftward divergence from the null line on modeled versus observed conditional quantile-quantile (Q-Q) plots.

To improve the discovery of genetic variants associated with SCZ and OCD, respectively, we used the condFDR method that incorporates genetic association summary statistics from the primary trait of interest together with a secondary phenotype. We then conducted conjFDR, an extension of the condFDR, to identify SNPs jointly associated with SCZ and OCD. Manhattan plots were constructed based on the conjFDR value to show the genetic risk loci shared between SCZ and OCD. Before the conjFDR analyses, SNPs in the complex LD region (extended MHC region: chr6:25119106-33854733; 8p23.1: chr8:7200000-12500000; MAPT region: chr17:40000000–47000000; apolipoprotein E region: chr19:44909039–45912650) were excluded^53^. We used 1000 Genomes Project Phase 3^54^ European sample as the LD reference (https://www.internationalgenome.org/data) and set a condFDR level of 0.01 and a conjFDR of 0.05 for comparison.

### Loci definition and functional annotation

We identified independent significant SNPs independent of each other at *r*^*2*^ < 0.6 and that reached a conjFDR<0.05 according to the FUMA protocol. Lead SNPs were selected using linkage equilibrium with each other at *r*^*2*^ < 0.1 were then as. We merged all loci which were less than 250 kb apart to define distinct genomic loci and selected the most significant P-value as a lead SNP of the merged locus.

We functionally annotated all candidate SNPs that were in LD (*r*^*2*^ ≥ 0.6) with one of the independent significant SNPs in the locus with Combined Annotation Dependent Depletion (CADD) scores^55^, RegulomeDB scores^56^, and Chromatin states scores^57,58^. We also linked candidate SNPs to genes using three gene-mapping strategies: positional mapping, expression quantitative trait locus (eQTL) mapping and chromatin interaction mapping. Then we conduct Gene Ontology (GO) to analyze gene-set enrichment for the mapped genes. All analyses were corrected for multiple comparisons. All LD information was calculated from the 1000 Genomes Project European-ancestry haplotype reference panel^59^.

### Mendelian randomization

We used two-sample Mendelian randomization (MR) to estimate the bidirectional causal association between SCZ and OCD.

Genetic instrumental variable (IV) was identified *(r*^*2*^ < 0.01 within a 10,000 kb window, *P* < 5 × 10^−8^). Fewer IVs were associated with OCD with genome-wide significance, so we relaxed the significance threshold (*P* < 1 × 10^−5^). Exposure IVs were extracted from the outcome GWAS. When a IV was missing in the outcome GWAS, we identified proxy variants using the LDLink online tool (*r*^*2*^ < 0.01 within a 10,000 kb window). We removed the weak IVs, defined as F-statistic < 10. Each SNP was detected in Phenoscanner (http://www.phenoscanner.medschl.cam.ac.uk/) to determine whether the estimate was violated by potential confounders. The inverse variance weighted (IVW) method was performed as the main analysis when valid IVs are valid or no pleiotropy. Moreover, simple mode, weighted mode, weighted median, and MR-Egger regression methods were used as sensitivity analyses. And we used MR Egger intercept and pleiotropy residual sum and outlier (MR-PRESSO) test^60^ to identify horizontal pleiotropic outliers. The statistical power of Mendelian randomization^61^ was calculated at http://cnsgenomics.com/shiny/mRnd/. The main statistical analyses were conducted using TwoSampleMR^62^ (Version: 0.4.22) and MR-PRESSO (Version: 1.0).

## Supplementary information

SUPPLEMENTAL MATERIAL

## Data Availability

All GWAS summary data sets used in the study areare publicly available (https://pgc.unc.edu/for-researchers/download-results/).
